# *Pseudomonas aeruginosa *in a neonatal intensive care unit: molecular epidemiology and infection control measures

**DOI:** 10.1186/1471-2334-9-70

**Published:** 2009-05-22

**Authors:** Valeria Crivaro, Anna Di Popolo, Alessandro Caprio, Antonietta Lambiase, Mario Di Resta, Tonia Borriello, Alda Scarcella, Maria Triassi, Raffaele Zarrilli

**Affiliations:** 1 Dipartimento di Scienze Mediche Preventive, Sezione di Igiene, Università Federico II, Via S Pansini 5, 80131 Napoli, Italy; 2 Dipartimento di Biologia e Patologia Cellulare e Molecolare, Università Federico II, Via S Pansini 5, 80131 Napoli, Italy; 3 Dipartimento di Pediatria, Università Federico II, Via S Pansini 5, 80131 Napoli, Italy

## Abstract

**Background:**

*Pseudomonas aeruginosa*, a non-fermentative, gram-negative rod, is responsible for a wide variety of clinical syndromes in NICU patients, including sepsis, pneumonia, meningitis, diarrhea, conjunctivitis and skin infections. An increased number of infections and colonisations by *P. aeruginosa *has been observed in the neonatal intensive care unit (NICU) of our university hospital between 2005 and 2007.

**Methods:**

Hand disinfection compliance before and after an educational programme on hand hygiene was evaluated. Identification of microrganisms was performed using conventional methods. Antibiotic susceptibility was evaluated by MIC microdilution. Genotyping was performed by PFGE analysis.

**Results:**

The molecular epidemiology of *Pseudomonas aeruginosa *in the NICU of the Federico II University hospital (Naples, Italy) and the infection control measures adopted to stop the spreading of *P. aeruginosa *in the ward were described. From July 2005 to June 2007, *P. aeruginosa *was isolated from 135 neonates and caused severe infections in 11 of them. Macrorestriction analysis of clinical isolates from 90 neonates identified 20 distinct genotypes, one major PFGE type (A) being isolated from 48 patients and responsible for 4 infections in 4 of them, four other distinct recurrent genotypes being isolated in 6 to 4 patients. Seven environmental strains were isolated from the hand of a nurse and from three sinks on two occasions, two of these showing PFGE profiles A and G identical to two clinical isolates responsible for infection. The successful control of the outbreak was achieved through implementation of active surveillance of healthcare-associated infections in the ward together with environmental microbiological sampling and an intense educational programme on hand disinfection among the staff members.

**Conclusion:**

*P. aeruginosa *infections in the NICU were caused by the cross-transmission of an epidemic clone in 4 neonates, and by the selection of sporadic clones in 7 others. An infection control programme that included active surveillance and strict adherence to hand disinfection policies was effective in controlling NICU-acquired infections and colonisations caused by *P. aeruginosa*.

## Background

Gram-negative bacteria have become relevant causes of healthcare-associated infections in the neonatal intensive care unit (NICU) environment. [1,2]. Outbreaks by Enterobacteriaceae have been widely described in this setting [3], and we recently reported the increased circulation of ESBL-producing *Klebsiella pneumoniae *and *Serratia marcescens *in the NICU of the Federico II University Hospital of Naples between 2002 and 2004 [4,5].

*Pseudomonas aeruginosa*, a non-fermentative, gram-negative rod, is responsible for a wide variety of clinical syndromes in NICU patients, including sepsis, pneumonia, meningitis, diarrhea, conjunctivitis and skin infections [6]. Nevertheless, if compared to other gram-negative bacteria, outbreaks by *P. aeruginosa *in NICU settings have been much less reported up-to-date and have been associated with both environmental reservoirs and healthcare workers' carriage [7-10].

An increased number of infections and colonisations by *P. aeruginosa *has been observed in the NICU of our university hospital between 2005 and 2007. The aims of this study were: i) to analyze the molecular epidemiology and antimicrobial susceptibility patterns of *P. aeruginosa *isolates; ii) to describe the infection control measures undertaken to limit *P. aeruginosa *spread in the ward.

## Methods

### Setting and surveillance measures

The tertiary-level NICU of the University 'Federico II' hospital of Naples, Italy serves approximately 350 admissions per year including both inborn and outborn patients and consists of three rooms with a maximum capacity of eight neonates per room. Sinks, chlorhexidine/alcohol hand disinfectants and gloves are available in each room. Sinks, 4% chlorhexidine hand disinfectants, and gloves are available in each room, together with hand disinfection instructions for staff members and visiting parents. Healthcare-associated infections were defined using standard Centers for Disease Control and Prevention definitions adapted to neonatal pathology [11]. Infections occurring after 48 hours of hospital stay were assumed to be hospital-acquired, those resulting from passage through the birth canal or from transplacentar transmission were excluded. For this study purposes, only severe infections (sepsis, meningitis, arthritis, pneumonia, urinary tract infections) were considered. Surveillance swabs from the nose/pharynx and rectum of each neonate admitted to the ward were analysed weekly. Informed consent to participate in this study was obtained from patients' parents. The study protocol was reviewed and approved by the local ethics committee. *P. aeruginosa *isolated from surveillance swabs and from clinical samples (blood, liquor, respiratory secretions, urine) of babies in the NICU were included in the study. Environmental samples were obtained from the following sites: air, room surfaces, sinks, hand disinfectants, baby incubators, monitors, and staff hands. All three sinks present in the ward were sampled.

### Hand disinfection educational programme

An educational programme on hand disinfection was repeatedly performed as part of the ward's plan for healthcare-associated infections control. It involved both medical and nursing staffs, it was carried out each time for one week, and it consisted of a 30 minutes review of the main topics on hand disinfection according to the US Centers for Disease Control and Prevention (CDC) recommendations [12]. Hand disinfection reminders were placed near each sink.

### Hand disinfection compliance evaluation and statistical analysis

Hand disinfection compliance was defined as hand-washing with the appropriate quantity of 4% chlorhexidine disinfectant and water for the recommended time, before and after each patient contact and it was evaluated by carrying out an observational study. Moreover, surveillance cultures from 30 randomly selected healthcare workers' hands were obtained by means of contact plates before and after each educational programme.

A trained, disguised observer monitored staff's (doctors and nurses) compliance to hand disinfection for the two weeks preceding and the two weeks following each series of educational meetings. Four patients were randomly selected each day (Monday through Friday) and all healthcare workers (HCWs) who cared for the target neonates were observed for 1 hour period during morning shifts and their compliance to hand disinfection procedures before and after each patient contact was recorded on a dedicated form.

Data were analysed using SPSS 11.0 (Chicago, IL, USA). Pearson's Chi-squared test was used to compare hand hygiene compliance before the beginning of the educational programme and after the first and the last series of educational meetings. Pearson's correlation coefficient between hand disinfection compliance in the same periods and colonisation rates was also calculated. All results were considered to be statistically significant at p < 0.05.

### Microbiological methods

Isolates were identified using the API NE manual identification system (bioMérieux, Marcy-L'Etoile, France) or the Phoenix automatic system (Becton Dickinson Bioscience, Spark, MD, USA). The susceptibililty of isolates to 12 selected antimicrobials was determined by the standard antimicrobial susceptibility methods [13]. The antimicrobials were: amikacin, aztreonam, cefepime, cefotaxime, ceftazidime, ciprofloxacin, gentamicin, imipenem, levofloxacin, meropenem, piperacillin, and piperacillin/tazobactam.

### Pulsed field gel electrophoresis

DNA macrorestriction with *Xba*I enzyme and pulsed-field gel electrophoresis (PFGE) was performed on all *P. aeruginosa *infection isolates and on available surveillance culture isolates as previously described [14].

## Results

### Epidemiology and Microbiological Features of *Pseudomonas aeruginosa*

**Figure 1:**
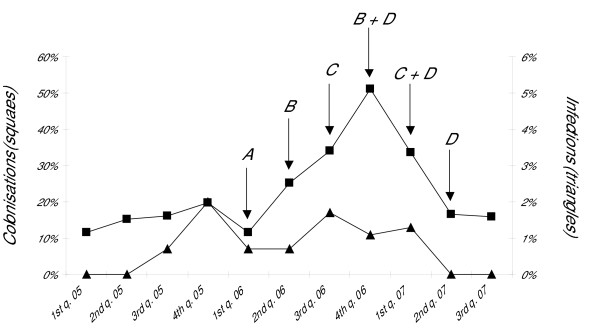
**Timing of control measures and incidence of isolation of *P. aeruginosa *in the NICU during the study period**. Squares and triangles represent neonates colonised or infected by *P. aeruginosa*, respectively. The following letters indicate the infection control interventions performed: A, alert surveillance for *P. aeruginosa*, collection of strains isolated from clinical samples, and reinforcement of contact isolation precautions; B, environmental microbiological sampling; C, reporting of PFGE analyses to staff members; D, thirty minutes daily educational programme on hand disinfection carried out in the ward for one week.

In the two years preceding the outbreak period *P. aeruginosa *has circulated in the ward in an endemic fashion, being responsible for a mean colonisation rate of 10.6% and for two ocular infections (data not shown). During the study period (July 2005 to June 2007), 616 neonates were prospectively surveilled for healthcare-associated infections (90.1% of total admissions to the ward) and 568 of them had their weekly surveillance swabs taken at least once. One hundred-thirty-five neonates (23.8% of all the microbiologically surveilled) became colonised by *P. aeruginosa *during their stay in the ward (Figure 1). Moreover, 72 patients developed 91 severe healthcare-associated infections, with an infection rate of 14.8%, corresponding to 5.94 severe infections/1000 patient-days. During the first year of the study (July 2005 to June 2006), 9.17 severe infections/1000 patient-days were registered, while such rate decreased to 3.24 severe infections/1000 patient-days during the second year (July 2006 to June 2007). During the study period, *P. aeruginosa *proved to be the third most common pathogen responsible for severe infections (12.1%), after *Candida spp*. (21.8%) and *Escherichia coli *(16.8%). Moreover, no pathogen was identified in 18.8% of infants diagnosed as having an infection. *P. aeruginosa *was responsible for 11 severe infections in 11 neonates, with 3 and 4 of them having their birth-weights below 1000 and 750 grams, respectively (Figure 1 and Table 1). All of the four neonates weighing less than 750 grams died; three of them died within the first 24 hours from *P. aeruginosa *infection diagnosis and one died after 11 days. In addition to isolation of *P. aeruginosa *from clinical samples (blood, respiratory secretions or urines), all 11 infected patients had positive cultures for *P. aeruginosa *at nasal/pharynx or rectum surveillance swabs.

**Table 1:** Clinical and microbiological features of *P. aeruginosa *isolates.

patient	gestational age (weeks)	birthweight (grams)	type of infection	outcome	resistance phenotype				antibio type	PFGE type
					TZP	IPM	GEN	CIP		
							
1	33,0	1950	sepsis	discharge	S	S	S	S	1	B
2	26,2	790	pneumonia	discharge	S	S	S	S	1	L
3	38,4	3160	pneumonia	discharge	S	S	S	S	1	A
4	24,4	700	sepsis	death	R	R	R	R	3	G
5	26,4	600	sepsis	death	S	R	R	R	2	O
6	28,0	960	pneumonia	discharge	S	S	S	S	1	K
7	36,0	2550	pneumonia	discharge	S	S	S	S	1	F
8	25,2	770	pneumonia	discharge	S	S	S	S	1	A
9	23,1	700	sepsis	death	S	S	S	S	1	A
10	33,0	1400	U.T.I.	discharge	S	S	S	S	1	R
11	30,0	650	pneumonia	death	S	S	S	S	1	A

NOTE. U.T.I., Urinary tract infection; S, susceptible; R, resistant.

**Figure 2:**
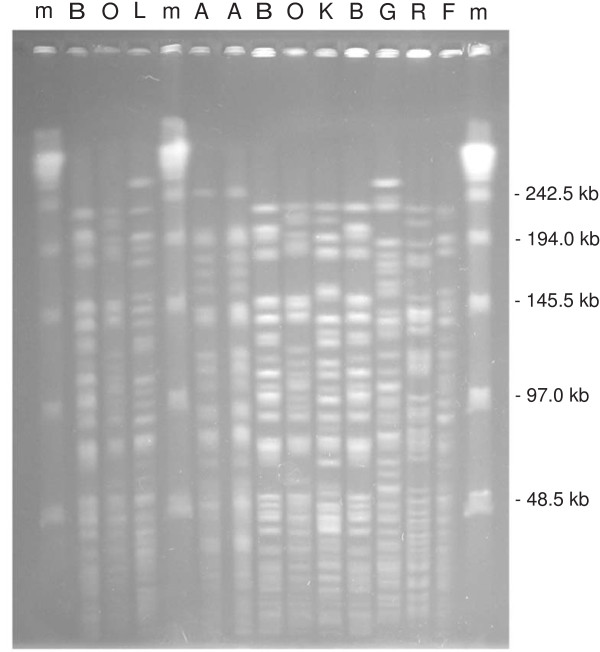
**Representative PFGE profiles of *P. aeruginosa *isolates from neonates in the NICU**. Capital letters on the top of the lanes indicate PFGE types identified; m, phage lambda DNA molecular mass markers. Sizes of lambda DNA molecular mass markers are shown on the right of the panel.

Molecular typing of 90 non-repetitive isolates including 79 available colonisation strains and all 11 infection strains (66.6% of all *P. aeruginosa *isolates) identified twenty PFGE types, named A through T, which showed up to six fragments variation in macrorestriction pattern (Figure 2 and data not shown). One predominant PFGE profile (type A) was identified in 48 strains from 48 different patients (53.3% of all typed isolates). This profile, which was responsible for 4 infections and 44 colonisations, first appeared in the ward in July 2005 and circulated until April 2007. Other recurrent profiles were Q, R, J and G, which were isolated in 6, 6, 5, and 4 patients, respectively, and caused no infections, with the exception of profile G (Table 1). Such PFGE profiles circulated in the ward in the following months: PFGE profile Q was isolated from August to September 2005, profile R during January and February 2007, profile J in December 2005, February 2006, January 2007 and May 2007, profile G during August, October and December 2005 and May 2007. The other six infections were caused by sporadic PFGE types (B, F, K, L, O, and R) (Table 1 and Figure 2). In all 11 infected patients, surveillance cultures and clinical samples showed identical PFGE profiles, thus excluding the possibility of multiclonal infection in the same neonate (data not shown).

With the exception of G and O PFGE profiles, all isolates recovered from infected neonates proved to be susceptible to aminopenicillins, ureidopenicillins, monobactams, second-, third- and fourth-generation cephems, carbapenems, fluoroquinolones, while resistant to trimethroprim-sulphometossazole, cloramphenicols and tetracycline. PFGE types G and O showed resistance also to imipenem and meropenem (MIC > 8), gentamicin (MIC > 8) and ciprofloxacin (MIC > 2); in addition, PFGE type G appeared to be resistant to piperacillin/tazobactam (MIC > 64/4). Moreover, such resistant profiles were responsible for two sepsis in two neonates with extremely low birth-weight, both having a fatal outcome (Table 1).

### Infection Control Interventions

After the sudden increase of infections caused by *P. aeruginosa *during the fourth quarter of 2005, a combination of targeted infection control measures were undertaken (Figure 1). A major concern of the infection control team was the appearance of a multi-drug resistant *P. aeruginosa *phenotype in two of the three infections which took place between October and December 2005 (Table 1).

During the first quarter of 2006 alert surveillance for *P. aeruginosa *was started and contact isolation for colonised and infected patients was reinforced. Moreover, all *P. aeruginosa *strains isolated from clinical samples and available strains isolated from surveillance swabs were collected for genotyping. After such control measures were implemented, infection rate slightly decreased, while the rate of colonised patients progressively increased.

Although no further isolations of multi-drug resistant *P. aeruginosa *were made, tighter control interventions were then undertaken owing to the marked increase of colonisations. Environmental microbiological sampling was performed twice during the outbreak period (Figure 1). Reporting of PFGE analysis results was followed by a 30 minutes daily educational programme on hand disinfection. Such control measures were periodically repeated as shown in Figure 1. The daily educational programme on hand disinfection was not specific for *P. aeruginosa *containment as it was part of the ward's plan for healthcare-associated infections control. Staff's overall attendance rate was high for all the educational programme's editions (> 90%).

Environmental microbiological sampling identified *P. aeruginosa *at the following sites: three sinks on both occasions and a nurse's hand on the second sampling. Genotyping of such strains demonstrated that the isolate recovered from one of the sinks on the first environmental sampling displayed an A profile and the one from the nurse's hand on the second environmental sampling displayed a G profile. The other environmental isolates showed different profiles, not corresponding to any of the profiles isolated from patients' surveillance swabs or clinical specimens.

No further targeted control measures were undertaken after June 2007 as no other infections by *P. aeruginosa *were diagnosed and colonisation rate returned to pre-epidemic values.

### Hand disinfection compliance evaluation

*P. aeruginosa *of PFGE profile G was isolated only once from surveillance cultures of a total of 90 randomly selected healthcare workers' hands before and after each educational programme, as described above. In addition, other 16 surveillance cultures proved to be inappropriate (according to the local infection control committee inappropriateness was defined as bacterial counts > 0.5 CFU/cm^2 ^in high risk wards). Hand disinfection compliance of HCWs before the first educational programme proved to be 23.4% and 11.7% before and after each patient contact, respectively (Table 2). Such rates significantly increased to 43.6% (p = .000) and 39.6% (p = .000), respectively after the first intervention (Table 2). Further significant increases were recorded after the last educational programme to 63.7% (p = .000) and to 57.1% (p = .000), respectively (Table 2). Improvement in hand disinfection compliance before patient contact proved to be very strongly, significantly, and inversely correlated with rates of *P. aeruginosa *colonisation (r = -1, p = .004, data not shown). Conversely, no significant correlation was found between the latter and improvement in hand disinfection compliance after patient contact (r = -.992, p = .081, data not shown). Finally, hand disinfection compliance of HCWs before and after patient contact proved to be significantly different at all times of observation (Table 2 and data not shown).

**Table 2:** Compliance with hand hygiene procedures in the NICU during the study period.

Hand disinfection observation period	Hand disinfection opportunities (n)	Compliance before opportunity (%)	Compliance after opportunity (%)
Prior to educational programme held in 4th quarter 2006	290	23.4	11.7
After educational programme held in 4th quarter 2006	250	43.6	39.6
After educational programme held in 2nd quarter 2007	226	63.7	57.1

## Discussion

The overall incidence rate of *P. aeruginosa *infections in NICUs is reported to be of approximately 10% [1,2,15], while infection attack rates during outbreak periods appear to be lower, ranging between 1% and 2.8% [10,16]. Higher attack rates have been reported [9,17], but such studies did not consider infections alone and analysed the combination of infections and colonisations. At our NICU, 11 neonates developed 11 *P. aeruginosa *severe infections, with an infection attack rate of 1.8%, and nearly 24% of the patients became colonised by *P. aeruginosa*, with an epidemic peak of 50% at 18 months from onset. Ten of the 11 infected patients were pre-term neonates (gestational age < 37 weeks) and 7 of them were of extremely low birth weight (ELBW), i.e. below 1000 grams. ELBW neonates have actually been shown to have a significantly increased risk of acquiring *P. aeruginosa *when compared to higher birth-weight infants [9]. Moreover, all of the four infected neonates who died weighed less than 1000 grams, therefore the crude mortality rate among ELBW patients infected by *P. aeruginosa *was of 57%. Although no attributable mortality rate was calculated, three of the four neonates died soon after (0–24 hours) the infection was diagnosed, thus our data indirectly confirm other Authors' findings regarding the very high mortality rates related to *P. aeruginosa *infections [15], especially in the lowest birth-weight categories' infants [18]. In our setting, in addition to the ELBW condition, two *P. aeruginosa *antibiotypes, both displaying resistance to imipenem, meropenem, gentamicin and ciprofloxacin, have probably affected the final outcome in two of the four fatal cases. We did not analyse mechanisms of resistance as no further such phenotypes were identified. Nevertheless, to our knowledge, this is one of the first accounts on two carbapenem-resistant *P. aeruginosa *genetically unrelated strains which caused two sepsis in a NICU.

*P. aeruginosa *frequently causes multi-clone outbreaks, with the concurrent isolation of genetically distinct strains among patients and healthcare workers (HCWs) and in the environment. During a 15 months-long epidemic in a NICU, Moolenaar et al. [9] identified the three main *P. aeruginosa *genotypes A, B, and C, isolated in 75%, 15%, and 10% of case-patients, respectively. Such genotypes were also found on three nurses' hands, while two positive environmental isolates showed two distinct genotypes, unrelated to any human isolate. Moreover, Foca et al. [17] described the circulation of multiple *P. aeruginosa *PFGE profiles over a 33 months-period in the NICU, showing the presence of a major clone, which was also isolated from the hands of a nurse, of two other PFGE types, and of eight unique clones. No environmental specimen proved to be positive for *P. aeruginosa*. Our study, covering a 24 months time span, identified a predominant PFGE type which was responsible for 36% of infections by *P. aeruginosa *and at least 35% of colonisations by the same pathogen. Such PFGE profile was also found in one sink, but not on any nurse's hand and circulated in the ward together with less recurrent and with sporadic strains, which caused the remaining infections and colonisations. Other five environmental samples proved to be positive for distinct *P. aeruginosa *PFGE types, unrelated to the ones colonising or infecting the patients. Transmission of *P. aeruginosa *from environmental sources to patients and HCWs has been thoroughly described [19,20]. Our findings indicate the presence in our NICU of multiple and distinct *P. aeruginosa *reservoirs, both environmental and human, and, owing to the long time period between the appearance in neonates (July 2005) and the environmental isolation (2^nd ^quarter 2006), we are not able to understand whether the only sink sample found to be positive for *P. aeruginosa *PFGE profile A has been a result, rather than the origin, of the pathogen's circulation in the ward.

Healthcare workers' (HCWs) hands have been frequently implicated in the spreading of *P. aeruginosa *in the NICU setting [9,10,17]. At our institution one HCW, with short to medium-length natural fingernails, had a positive hand culture for *P. aeruginosa*, displaying the G genotype. The culture was drawn when the epidemic was at its peak value, with nearly 50% of neonates being colonised. The detection of HCWs who are colonised at any body site by *P. aeruginosa *epidemic clones may sometimes identify the pathogen's reservoir, thus enabling a successful and timely outbreak containment [10]. Owing to organizational difficulties, the HCW found to have a positive hand culture at our NICU could not be reassigned to non-clinical activities. Moreover, no further analyses have been performed to establish her contribution to the outbreak, therefore we can only hypothesize that she has been a transient carrier of one of the less recurrent epidemic clones. Actually, patient exposure within the first 14 days of NICU admission to a HCW with short natural fingernails and with one positive hand culture for a *P. aeruginosa *epidemic clone has not been recognized as an independent risk factor for acquiring *P. aeruginosa *colonization or infection [9]. In turn, exposure to two HCWs with negative hand cultures has been associated with an increased risk of colonisation by a *P. aeruginosa *epidemic clone on multivariate analysis [17]. This finding suggests that transient colonisations of HCWs' hands by *P. aeruginosa *may be underestimated during outbreaks investigations and that reinforcement of hand disinfection and of correct gloves use should always be promptly initiated when an increased number of *P. aeruginosa *isolations is detected. In agreement with previous data [21], our study shows that, compared with all the traditional infection control interventions undertaken to contain *P. aeruginosa *circulation of in the ward, the hand disinfection educational programme was the most effective one. The programme started during the fourth quarter of 2006, when the outbreak was at its peak value, and by the end of the second quarter of 2007 the outbreak was over. Owing to the programme's success, the meaning of different hand disinfection compliance rates before and after patient contact and how this may have affected *P. aeruginosa *circulation in the ward were not further investigated. A possible explanation for the partial ineffectiveness of the traditional infection control interventions may be the molecular heterogeneity of *P. aeruginosa *outbreaks. Thus, the timely identification of increased isolation of this pathogen, achieved by means of active surveillance, appears to be crucial to limit the spreading of *P. aeruginosa *in NICU settings.

## Conclusion

This study suggests that an infection control programme based on active surveillance and strict adherence to hand disinfection and gloves use policies and supported by environmental sampling and molecular analysis is effective in controlling NICU multi-clone *P. aeruginosa *outbreaks.

## Competing interests

The authors declare that they have no competing interests.

## Authors' contributions

VC, AS, AC and MDR carried out the active surveillance of healthcare-associated infections and infection control interventions in the NICU, AL, ADP and TB isolated the *P. aeruginosa *strains and carried out the antimicrobial susceptibility experiments, ADP performed the PFGE experiments, VC, MT and RZ conceived the study and participated in its design and coordination, VC and RZ drafted the manuscript. All authors read and approved the final manuscript.

## Pre-publication history

The pre-publication history for this paper can be accessed here:

http://www.biomedcentral.com/1471-2334/9/70/prepub
